# The clinical characteristics of PFAPA syndrome

**DOI:** 10.1186/1546-0096-12-S1-P250

**Published:** 2014-09-17

**Authors:** Sara Sebnem Kilic

**Affiliations:** 1 Pediatric Rheumatology, Uludag University Medical Faculty, Bursa, Turkey

## Introduction

The periodic fever, aphthous stomatitis, pharyngitis and adenitis (PFAPA) syndrome is considered as a non-inherited autoinflammatory disease of unknown etiology. The most common clinical manifestations are high fever episodes with clockwork periodicity every 3 to 8 weeks and lasts for about 3 to 6 days, associated to pharyngitis, aphthous stomatitis, and cervical adenitis.

## Objectives

We described the clinical and laboratory outcomes of 149 pediatric patients diagnosed with PFAPA. The clinical response to colchine was evaluated.

## Methods

Hundered and fourty-nine patients [ 58 female (38,9 %) and 91 male (61,1 %)] with PFAPA Syndome were included in this study. The following tests were applied at the onset of a febrile episode; complete blood count, erythrocyte sedimentation rate, CRP, Serum Amiloid A, Procalcitonin, serum immunoglobulins (IgG, IgM, IgA) and FMF mutations (MEFV gene).

## Results

Individual episodes of fever usually resolved with a single intramuscular 1 mg/kg dose of methyl prednisolone. The mean fever resolve duration during the febril episode was 1.64 hours. FMF gene was investigated in 109 and we determined heterozygot MEFV gene mutations in 19 of our patients (17%). At the onset of febril episodes the mean CRP value was 6.09±8.48 mg/dl (1-38), the mean Serum Amiloid A value was 240 mg/L (2,7-1900), Prokalsitonin values were negative. Our testing included at least a CBC at the onset of febril episodes (the mean WBC: 13150/mm3, range 4500-26.400 cells/mm³) with a preponderance of neutrophils. IgM levels were low in the 5 patients, IgA levels were low in the 6 patients and IgG levels were low 11 patients according to their healthy age matched controls. PFAPA patients had normal CRP and Procalsitonin levels between febril episodes. A total of 89 patients were treated with colchicine. The mean interval between episodes was statistically prolonged in patients who were on prophylactic colchicine therapy.

## Conclusion

Both pediatricians and ENT specialist have to keep PFAPA Syndrome in their mind when they encounter with a patient with recurrent fever attacks before starting antibiotics. The colchicine treatment was found effective in decreasing the frequency of fever episodes.

## Disclosure of interest

None declared.

